# Neutrophilic asthma: advances in experimental models from pathogenesis to therapeutic translation

**DOI:** 10.1186/s10020-026-01507-3

**Published:** 2026-06-01

**Authors:** Jialin Yao, Jingjing Lu, Jing Gao, Ximing Liao, Di Wu, Muyun Wang, Qiang Li, Wei Gao

**Affiliations:** Department of Pulmonary and Critical Care Medicine, Shanghai East Hospital, School of Medicine, Tongji University, No.150 Jimo Road, Pudong, Shanghai, 200092 People’s Republic of China

**Keywords:** Neutrophilic asthma, Experimental models, Th17 inflammation, Neutrophil extracellular traps, Steroid resistance

## Abstract

**Background:**

Neutrophilic asthma (NA) is a severe and therapy-resistant asthma endotype marked by persistent airway inflammation, neutrophil infiltration, and limited response to glucocorticoids. The development of targeted therapies has been hampered by insufficient mechanistic insight and the lack of physiologically relevant experimental models.

**Main body:**

This review comprehensively synthesizes current in vivo and in vitro models for NA, discussing their methodologies, mechanistic fidelity, and translational potential. Murine systems provide flexible genetic and induction platforms; rat models reliably replicate steroid resistance; and equine models spontaneously mimic chronic human disease. Advanced in vitro approaches including air–liquid interface cultures, patient-derived organoids and lung-on-a-chip devices, enable precise study of epithelial barrier dysfunction, immune crosstalk, and personalized drug responses.

**Conclusion:**

By critically evaluating these tools, our review establishes a foundation for model standardization and underscores their collective role in elucidating NA pathogenesis and advancing precision medicine.

**Graphical Abstract:**

**Figure:**
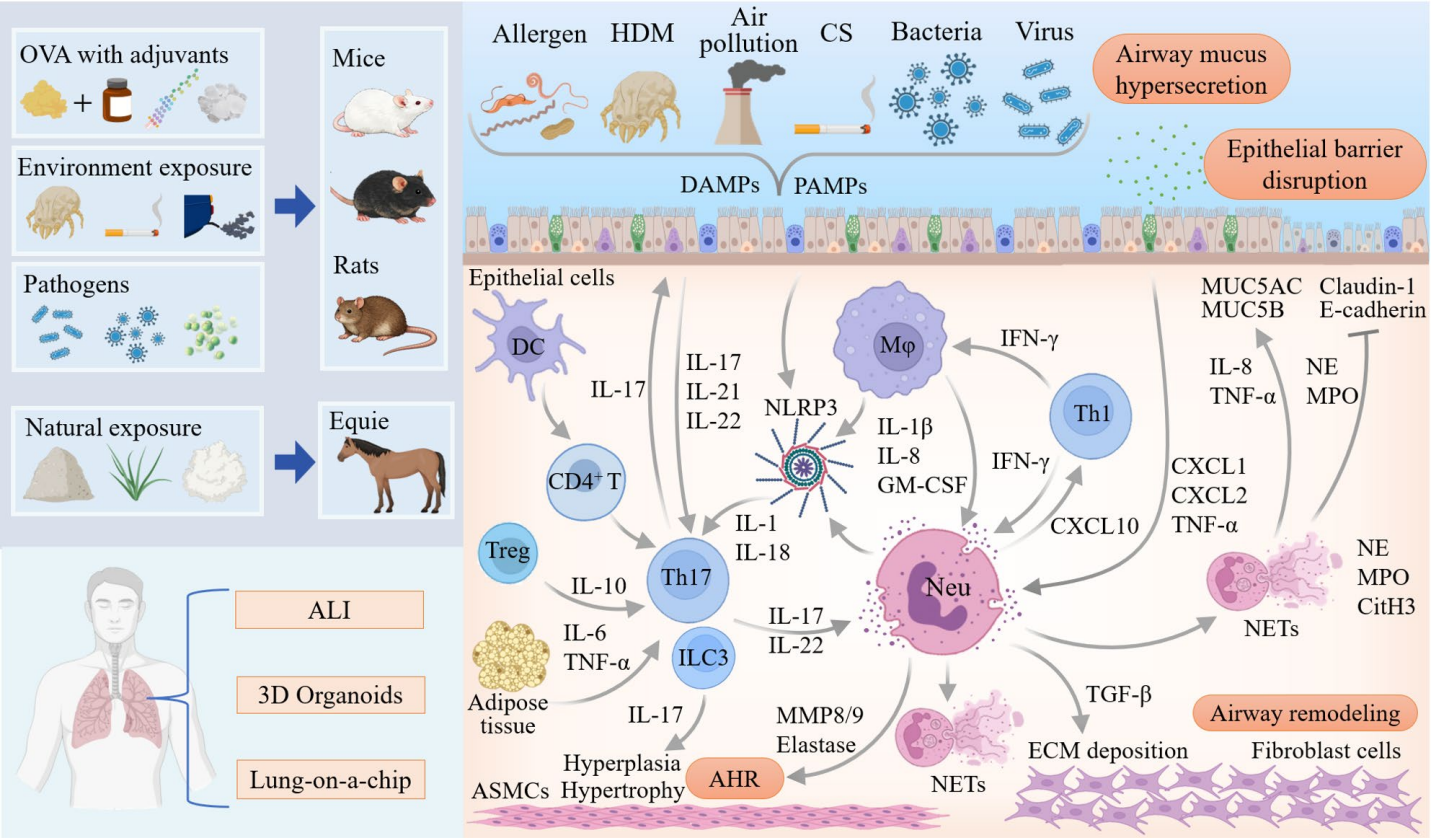
This review illustrates the major experimental platforms for modeling neutrophilic asthma, including murine, rat, equine, air-liquid interface, organoid, and lung‑on‑a‑chip systems. These models recapitulate key hallmarks, such as Th17‑driven inflammation, neutrophil recruitment and activation, epithelial barrier disruption, inflammasome activation, airway remodeling, and glucocorticoid resistance.

## Introduction

Asthma is a heterogeneous respiratory disease characterized by chronic airway inflammation, airway hyperresponsiveness (AHR), and airflow limitation, affecting over 300 million people globally (Jayasooriya et al. 2025; Oh et al. 2025). It is broadly classified into Type 2 (T2)-high and T2-low phenotypes based on immune mechanisms (Gyawali et al. 2025). T2-high asthma involves eosinophilic inflammation driven by interleukin (IL)−4, IL-5, IL-13, and immunoglobulin E, and typically responds to glucocorticoids (Lommatzsch et al. 2025). In contrast, T2-low asthma remains poorly understood and therapeutically challenging (Liu et al. 2024). Neutrophilic asthma (NA), a T2-low subtype, is defined by sustained sputum neutrophilia, poor glucocorticoid responsiveness, late-onset severe disease, comorbidities such as smoking and obesity, and frequent exacerbations triggered by infections or environmental irritants rather than allergens (Peri et al. 2023; He et al. 2025; Chung et al. 2014). Pathologically, NA involves Th1/Th17 inflammation, neutrophil infiltration, neutrophil extracellular traps (NETs) formation, and cytokines like IL-17, IL-6, and tumor necrosis factor-alpha (TNF-α) (Lourenco et al. 2022). Emerging evidence also highlights inflammasome, particularly NLRP3 activation and disruption of epithelial tight junction proteins as additional hallmarks linked to steroid resistance and disease severity (Tan et al. 2019).

NA research relies heavily on animal models, primarily mice, using stimuli such as ovalbumin (OVA) plus lipopolysaccharide (LPS) or house dust mite (HDM) (Chen et al. 2025; Woodrow et al. 2023). However, substantial methodological variations, especially in sensitization routes, challenge protocols, dosing, and duration, hinder their reproducibility and consensus (Woodrow et al. 2023; Russjan And Kaczynska 2018). Although previous reviews have synthesized the role of neutrophils in steroid‑resistant asthma or broadly cataloged animal models for multiple asthma phenotypes (Nabe 2020; Hudey et al. 2020), a dedicated, mechanism‑centric systematic evaluation of NA models, along with practical guidance for model selection, remains lacking. This review directly addresses this gap by critically appraising in vivo and in vitro NA modeling strategies, focusing on how different models recapitulate specific clinical and pathophysiological features, covering allergen selection, species differences, experimental procedures, and phenotypic validation. Our goal is to provide a framework for rational model selection tailored to specific research goals, thereby bridging mechanistic discovery and therapeutic translation, standardizing research, and supporting the development of new therapeutic approaches.

## The role of neutrophil-driven inflammation in NA

Neutrophils are innate immune cells produced in the bone marrow under the control of granulocyte colony-stimulating factor (Crisford et al. 2021). They contain three granule types (azurophilic, specific, gelatinase) and are recruited to airways primarily by chemokines such as leukotriene B4, IL‑8, and C-X-C motif chemokine ligand (CXCL) 1 (Aroca-Crevillen et al. 2024). Once activated, neutrophils eliminate pathogens via phagocytosis, degranulation, or NET formation (NETosis) (Gao et al. 2025). They also release pro-inflammatory cytokines to recruit other immune cells or modulate immune responses via anti-inflammatory factors.

In NA, neutrophil-driven mechanisms contribute to disease severity and steroid resistance (Kalchiem-Dekel et al. 2020). Environmental triggers, like tobacco smoke, pollution, and chronic infections, damage airway epithelium, upregulate Toll-like receptor (TLR) 2/TLR4, and promote Th1/Th17 polarization (Sze et al. 2020). Th1 cells secrete interferon-gamma (IFN-γ), enhancing macrophage cytotoxicity and neutrophil recruitment. Th17 cells produce IL‑17A (often synergizing with IL‑22/IL‑21), driving neutrophil infiltration and activation, and stimulating airway epithelial cells (AECs) to release IL‑8 and granulocyte–macrophage colony-stimulating factor (Reza And Ambhore 2025). Macrophages amplify inflammation via IL‑1β and TNF‑α, prolonging neutrophil survival and elevating reactive oxygen species production (Specjalski et al. 2023).

NETs play a pivotal role in NA pathology. Upon NETosis, neutrophils release DNA scaffolds decorated with elastase and myeloperoxidase (Xiang et al. 2025). NETs degrade E-cadherin and tight junction proteins, disrupting epithelial integrity and increasing permeability (Hudock et al. 2022). Extracellular DNA interacts with mucus, promoting disulfide cross-linking and persistent mucus plugs (Keir And Chalmers 2022). NETs also activate the TLR4/nuclear factor-kappa B (NF-κB) pathway, inducing AECs to secrete CXCL1, CXCL2, and CXCL8, which recruit more neutrophils via C-X-C chemokine receptor (CXCR) 1/CXCR2 (Keir et al. 2021; Tsai et al. 2023). Besides, NETs‑derived cytoplasts can activate dendritic cells (DCs), promoting Th17 differentiation and sustained IL‑17 production (Krishnamoorthy et al. 2018).

**Fig. 1:**
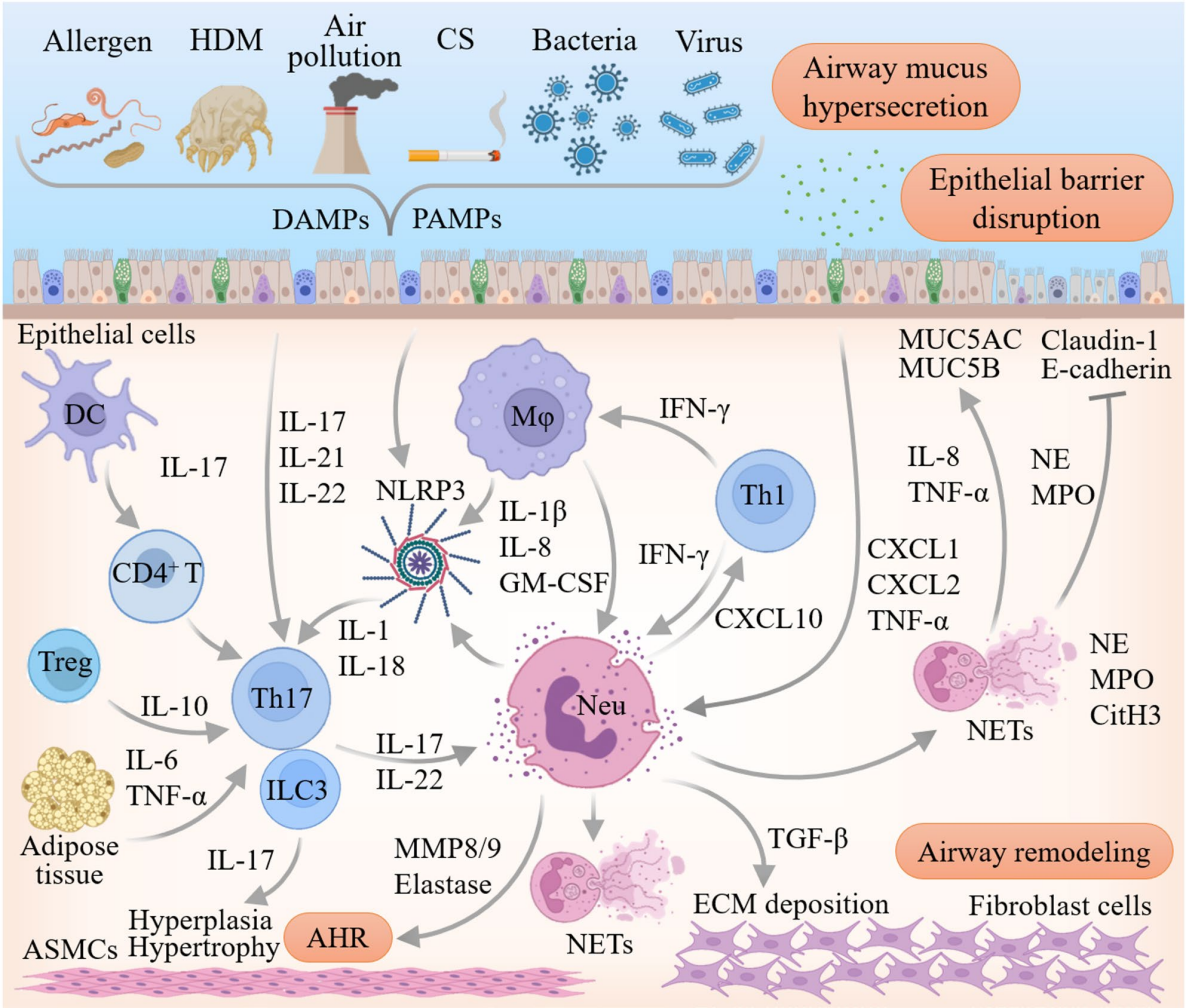
Mechanisms of NA. Allergens, environmental insults, and pathogens activate airway epithelial cells and innate immunity, promoting Th1/Th17 polarization and persistent neutrophilic inflammation. Pro-inflammatory mediators, like IL-17A, IL-6, IL-1β, and TNF-α drive neutrophil recruitment and activation. Activated neutrophils release ROS and NETs, resulting in epithelial barrier disruption, mucus hypersecretion, airway remodeling, AHR, and glucocorticoid resistance. Inflammasome activation and tight junction impairment perpetuate this inflammatory cycle. Abbreviation: AHR: airway hyperresponsiveness; ASMCs: airway smooth muscle cells; CS: cigarette smoke; CitH3: citrullinated histone H3; CXCL: C-X-C motif chemokine ligand; DAMP: damage-associated molecular pattern; PAMP: pathogen-associated molecular pattern; DC: dendritic cell; ECM: extracellular matrix; GM-CSF: granulocyte–macrophage colony-stimulating factor; ILC3: group 3 innate lymphoid cell; HDM: house dust mite; IFN-γ: interferon-γ; IL: interleukin; Mφ: macrophage; MMP: matrix metalloproteinase; MUC: mucin; MPO: myeloperoxidase; NE: neutrophil elastase; NETs: neutrophil extracellular traps; Treg: regulatory T cell; TGF-β: transforming growth factor-β; Th: T helper cell; TNF-α: tumor necrosis factor-α

Beyond NETs, the NLRP3 inflammasome further amplifies Th17‑driven neutrophilic inflammation, and in NA, it is activated by oxidative stress, bacterial products, or environmental pollutants (Williams et al. 2021). Upon activation, NLRP3 triggers caspase‑1‑dependent maturation of IL‑1β and IL‑18, which promote Th17 differentiation and neutrophil recruitment, while also contributing to glucocorticoid resistance (Kim et al. 2017). Moreover, Th17 cytokines, especially IL‑17A, downregulate tight junction proteins in AECs, increasing barrier permeability (Lässer et al. 2026). This disruption releases damage-associated molecular patterns that further activate the inflammasome, creating a self‑amplifying loop. Thus, measuring inflammasome activity and tight junction integrity provides valuable readouts for NA models and potential therapeutic targets. Together, these neutrophil‑related mechanisms are recapitulated across different NA models, guiding model selection and mechanistic research (Fig. 1).

## In vivo models of NA

### Murine models

Among in vivo NA models, the mouse system is most widely utilized due to genetic tractability and cost-effectiveness, typically employing C57BL/6 or BALB/c strains. Before detailing specific induction protocols, it is important to recognize that the duration of stimulus exposure critically determines which pathological features of NA are recapitulated in murine models. Specifically, acute protocols (typically 2–4 weeks) predominantly induce neutrophil infiltration, elevated Th17 cytokines, and AHR, but generally do not produce significant structural remodeling (Kim et al. 2020; Wen et al. 2024). Sub-chronic models (4–8 weeks) begin to show goblet cell hyperplasia, subepithelial collagen deposition, and early airway smooth muscle changes (Quoc et al. 2023). Chronic models (8–24 weeks) are required to mimic long-term features such as sustained steroid resistance, peribronchial fibrosis, airway smooth muscle hypertrophy, and mucus plugging (Li et al. 2020). For obesity-related NA, prolonged high-fat diet (HFD) feeding (10–18 weeks) is necessary to establish systemic metabolic dysfunction before airway challenge (Tashiro et al. 2017). Therefore, researchers should select model duration based on their specific endpoint: short durations for mechanistic studies of acute neutrophilic inflammation, intermediate durations for evaluating anti-inflammatory compounds, and chronic durations for assessing anti-remodeling or steroid-restoring therapies.

#### OVA combined with adjuvants-induced models

OVA, the major egg white protein, is a common model antigen (Liu et al. 2024). It is important to note that the classic OVA‑aluminum hydroxide (Alum) protocol (intraperitoneal sensitization with OVA adsorbed to aluminum hydroxide followed by OVA aerosol challenge) is the standard model for Th2‑driven eosinophilic asthma, characterized by elevated immunoglobulin E, IL‑4, IL‑5, IL‑13, and airway eosinophilia. However, by modifying the adjuvant, the dose, or the challenge regimen, OVA can also be used to induce NA with a Th17‑dominant, steroid‑resistant phenotype. In NA studies, the most frequently used adjuvants with OVA are Complete Freund’s Adjuvant (CFA), LPS, and less commonly, Alum when combined with LPS (Jia et al. 2024). Regarding the mechanism of adjuvants, CFA contains heat‑killed Mycobacterium tuberculosis, which activates TLR2/TLR4, and NOD2 receptors on antigen-presenting cells, triggering MyD88‑dependent NF‑κB signaling and promoting Th1/Th17 differentiation (Behr And Divangahi 2015). LPS, a TLR4 agonist, induces IL‑1β, IL‑6, and IL‑23 production via the MyD88 pathway, skewing the response toward Th17 and neutrophilic inflammation (Liu et al. 2023). Alum alone typically drives Th2 responses; however, when combined with high‑dose or repeated LPS challenge, it can shift toward a mixed Th2/Th17 or even Th17‑dominant phenotype. Thus, adjuvant choice determines the inflammatory endotype (An et al. 2022). Typical protocols use 6- to 8-week-old female mice. Sensitization consists of 2–4 injections (intraperitoneal or intranasal) of 10–100 μg OVA combined with 75 μL CFA, 1–10 μg LPS, or 1–2 mg Alum. Challenge is performed 3–5 times via aerosol or intranasal delivery with 0.1%−5% OVA, with or without 1–10 μg LPS, over 17–44 days (Table 1) (Kim et al. 2020; Wen et al. 2024; Pan et al. 2021). Crucially, the OVA-Alum protocol that induces Th2 inflammation with low‑dose, infrequent challenge can shift to a neutrophilic phenotype when challenge is intensified or when LPS is co‑administered. This distinction is often overlooked in the literature, and we explicitly highlight it here to guide model selection.

**Table 1:** Characteristics of NA mouse models induced by OVA combined with adjuvants

Mouse	Sensitization		Challenge		Duration	Subset	Related mechanism	References
Strain (Sex, Age)	Agents (Routes)	Frequency	Agents (Routes)	Frequency				
BALB/c (F, 4 weeks)	75 μg OVA + 10 μg LPS (i.n.)	Day 21, 22, 23, 28	50 μg OVA (i.n.)	Day 35, 36, 42, 43	44 days	NEU	IL-17/DEL-1 pathway	(Jia et al. 2024)
BALB/c (F, 6 weeks)	50 μg OVA + 75 μL CFA (i.p.)	Day 1, 2, 3	5% OVA (i.n.)	Day 14, 17, 21, 24, 27	28 days	TH1, TH17, NEU, DC	IL-17/IL-8 pathway	(Pan et al. 2021)
BALB/c (F, 6–8 weeks)	20 μg OVA + 75 μL CFA (i.p.)	Day 0, 7, 14	5% OVA (NEB)	Day 21–30	31 days	NEU, Mφ	Necroptosis pathway	(Zhang et al. 2024)
BALB/c (F, 6–8 weeks)	20 μg OVA (i.p.) 100 μL CFA/IFA (i.p.)	Day 0; Day14, 28	OVA + LPS (NEB)	Day 38–42	44 days	TH1/17, NEU, Mφ	TH1/17/IFN-γ/IL-17A/CXCR2 pathway	(Shilovskiy et al. 2024)
BALB/c (F, 6–8 weeks)	20 µg OVA + 75 µL CFA (i.p.)	Day 0, 7, 14	5% OVA (NEB)	Day 21–30	30 days	NEU, DC	Ferroptosis pathway	(Liao et al. 2024)
BALB/c (M, 20–25 g)	50 μg OVA + 6 mg Alum (i.p.)	Day 1, 14	10 mg/ml OVA (NEB); 0.1 mg/ml LPS (i.t.)	Day 22, 24, 26, 28; Day 28,	29 days	TH17, NEU, DC	TH17/IL-17/NF-κB pathway	(Camargo et al. 2017)
BALB/c (F, 6 weeks)	100 μg OVA + 1 mg Alum (i.p.)	Day 1, 7, 14	OVA + LPS (i.n.); 3% OVA (NEB)	Day 15, 17, 19; Day 21, 23, 25	27 days	NEU, Mφ	M1 polarization; autophagy; NETosis	(Quoc et al. 2023)
BALB/c (F, 4 weeks)	100 μg OVA + 10 μg LPS + 2 mg Alum (i.p.)	Day 0, 7, 14	5% OVA (i.n.)	Day 21, 22, 23	24 days	TH17	UBD/IL-17 pathway	(Liu et al. 2024)
C57BL/6 (F, 6–8 weeks)	20 μg OVA + 75 μL CFA (i.p.)	Day 0, 7	0.5% OVA (NEB)	Day 14, 15, 16	17 days	TH17, NEU, Mφ	IL-17A pathway; M1 polarization	(Wen et al. 2024)
C57BL/6 (F, 6–8 weeks)	20 μg OVA + 75 μl CFA (s.c.)	Day 0	0.1% or 1% OVA (INH)	Day 21, 22	23 days	TH1, TH17, NEU	IL-17 pathway	(Dejager et al. 2015)
C57BL/6 (F, 6 weeks)	20 μg OVA + 75 μl CFA (i.p.)	Day 0, 7, 14	0.1% OVA (i.n.)	Day 21, 22	23 days	TH17, NEU	TH17/IL-17 pathway	(Lee et al. 2017)
C57BL/6 (M/F, 6–8 weeks)	75 μg OVA + 10 μg LPS (i.n.)	Day 0, 1, 2, 7	50 μg OVA (i.n.)	2 days/week for 4 weeks	36 days	TH17, NEU	IL-17A, TNF-α/G-CSF pathway	(Kim et al. 2020)
C57BL/6 (M, 6–8 weeks)	75 μg OVA + 10 μg LPS (i.n.)	Day 0, 1, 2, 7	100 μg OVA (i.n.)	Day 14, 15, 21, 22	24 days	TH17, Mφ	IL-23/TH17/NETosis	(Han et al. 2024)
C57BL/6 (F, 6–8 weeks)	100 μg OVA + 0.1 μg LPS (OA)	Day 0, 6, 13	1% OVA (NEB)	Day 21, 22, 23	23 days	TH17, NEU	TH17/IL-7/JAK/STAT5 pathway; Bcl-2, caspase-3/IL-17 pathway	(Zhang et al. 2020)
C57BL/6 (M/F, 6–12 weeks)	50 μg OVA + 100 ng LPS (OA)	Day 0, 7	50 μg OVA (OA)	Day 14–16	17 days	NEU, Mφ	TLR/NRP2/efferocytosis	(Immormino et al. 2022)
C57BL/6 (F, 6–8 weeks)	75 μg OVA + 10 μg LPS (i.n.)	Day 0, 1, 2, 7	50 μg OVA (i.n.)	Day 14, 15, 21, 22	23 days	TH17, DC	IL-23/TH17 pathway	(Zhang et al. 2015)
C57BL/6 (F, 8–10 weeks)	75 μg OVA + 10 μg LPS (i.n.)	Day 0, 1, 2, 7	50 μg OVA (i.n.)	Day 14, 15, 21, 22	23 days	TH17, NEU, DC	RAGE/HMGB1/IL-23/TH17 pathway	(Zhang et al. 2017)
C57BL/6 (F, 6–8 weeks)	100 μg OVA + 1 μg LPS (OA)	Day 1, 7, 14	1% OVA (INH)	Day 21–27	27 days	NEU	HMGB1/RAGE/PI3K/ERK pathway	(Zhang et al. 2022)
C57BL/6 (F, 6 weeks)	100 μg OVA + 2 mg Alum (i.p.)	Day 0, 7	50 μg OVA (i.n.); 10 μg LPS (i.n.)	Day 14, 15, 21, 22, 23; Day 21, 22, 23	24 days	TH17, NEU	TH17/IL-17 pathway	(An et al. 2018)
C57BL/6 (F, 6 weeks)	100 μg OVA + 2 mg Alum (i.p.)	Day 0, 7	50 μg OVA (i.n.); 10 μg LPS (i.n.)	Day 14, 15, 21, 22, 23; Day 21, 22, 23	24 days	TH17, NEU	TH17/IL-17/IL-22 pathway; HDAC2 pathway	(Park et al. 2022)
C57BL/6 (F, 6 weeks)	100 μg OVA + 2 mg Alum (i.p.)	Day 0, 7	50 μg OVA (i.n.); 2 mg/kg LPS (i.n.)	Day 14, 15, 21, 22, 23; Day 18, 21, 23	24 days	NEU, TH17	IL-17A pathway	(An et al. 2022)

Representative OVA-based NA mouse models induced by CFA, LPS, Alum, or combined adjuvants. Parameters include mouse strain, age, sex, sensitization/challenge protocols, duration, inflammatory subsets, mechanisms, and references, to aid protocol comparison and model selection

*Abbreviation*: *Bcl-2* B-cell lymphoma 2, *CFA* Complete Freund's Adjuvant, *CXCR* C-X-C motif chemokine receptor, *DC* dendritic cell, *DEL-1* developmental endothelial locus 1 protein, *G-CSF* granulocyte colony-stimulating factor, *HMGB1* high mobility group box 1 protein, *HDAC2* histone deacetylase 2, *IFN-γ* interferon-γ, *IFA* Incomplete Freund's Adjuvant, *IL* interleukin, *IL-17A* interleukin-17A, *INH* inhalation, *JAK* Janus kinase, *LPS* lipopolysaccharide, *Mφ* macrophage, *M1* M1 macrophage, *NEB* nebulization, *NETosis* neutrophil extracellular trap formation, *NEU* neutrophil, *NRP2* neuropilin 2, *OA* oropharyngeal administration, *OVA* ovalbumin, *ERK* extracellular regulated protein kinases, *RAGE* receptor for advanced glycation end products, *i.n.* intranasal, *i.p.* intraperitoneal, *s.c.* subcutaneous, *STAT* signal transducer and activator of transcription, *TH1* T helper cell 1, *TH17* T helper cell 17, *TLR* toll-like receptor, *TNF-α* tumor necrosis factor-α, *UBD* ubiquitin D, *M* male, *F* female

**Fig. 2:**
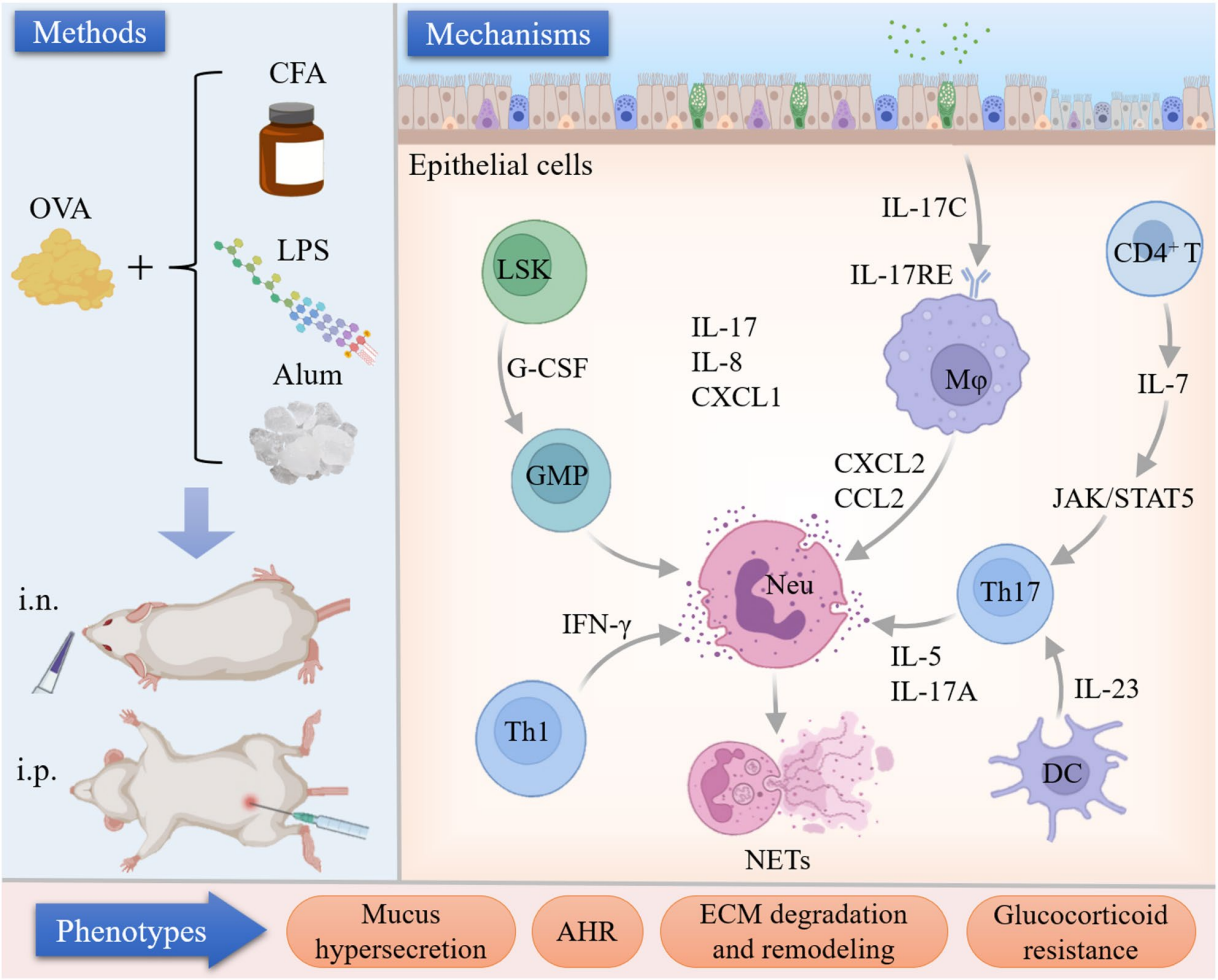
Methods and mechanisms of OVA with adjuvants-induced NA mouse models. OVA‑based NA models are established using adjuvants that promote Th1/Th17 responses, including CFA, LPS, or Alum combined with LPS. These adjuvants activate innate immune receptors, enhance antigen‑presenting cell function, and drive Th1/Th17 polarization. Abbreviation: AHR: airway hyperresponsiveness; Alum: aluminum hydroxide; CCL2: C–C motif chemokine ligand 2; CFA: complete Freund's adjuvant; CXCL: C-X-C motif chemokine ligand; DC: dendritic cell; ECM: extracellular matrix; G-CSF: granulocyte colony-stimulating factor; GMP: granulocyte-monocyte progenitor; IFN-γ: interferon-γ; IL: interleukin; IL-17RE: interleukin-17 receptor E; JAK/STAT5: Janus kinase/signal transducer and activator of transcription 5; LSK: Lineage⁻ Sca-1⁺ c-Kit⁺ cells; LPS: lipopolysaccharide; Mφ: macrophage; Neu: neutrophil; NETs: neutrophil extracellular traps; OVA: ovalbumin; Th: T helper cell

OVA‑induced NA models are primarily driven by Th17 pathway activation (Zhang et al. 2020). Adjuvants initiate and amplify this pathway: activated Th17 cells produce IL‑17A, stimulating lung epithelial cells, endothelial cells, and fibroblasts to generate neutrophil chemoattractants (CXCL1, CXCL5, IL‑8), driving robust neutrophil recruitment (Camargo et al. 2017; Shilovskiy et al. 2022). Meanwhile, IL‑17A synergizes with IFN‑γ and TNF‑α to amplify inflammation and enhance neutrophil cytotoxicity (Dejager et al. 2015). In CFA‑ or LPS‑augmented models, additional mechanisms include signal transducer and activator of transcription 1 activation via IL‑17C/IFN‑γ, S100A9‑induced IL‑1β release, and histone deacetylase 2 (HDAC2) downregulation leading to steroid resistance (Zhang et al. 2015; Zhang et al. 2017; Lee et al. 2017; Park et al. 2022). Together, these models recapitulate core human NA features, despite variations induced by distinct adjuvants. The most direct readout is a significant increase in neutrophil count and proportion in bronchoalveolar lavage fluid (BALF), distinguishing it from eosinophilic asthma (An et al. 2018). Correspondingly, Th17-related and pro-inflammatory cytokines are highly elevated in both BALF and lung homogenates (Quoc et al. 2023; Han et al. 2024). Histologically, lung tissues exhibit marked neutrophilic peribronchial/perivascular infiltration, airway wall thickening, subepithelial collagen deposition, and goblet cell hyperplasia (Park et al. 2022; Zhang et al. 2024; Zhang et al. 2022). These changes underlie increased methacholine-induced AHR, a hallmark of airway dysfunction (Immormino et al. 2022). A critical translational feature is steroid resistance, particularly in CFA- or LPS-augmented models, where glucocorticoids fail to reduce neutrophilic inflammation or AHR (An et al. 2022; Shilovskiy et al. 2024; Liao et al. 2024). In a high-dose OVA-adjuvant model, mixed granulocytic inflammation occurs; neutrophil depletion significantly attenuates the late asthmatic response, underscoring a key functional role of neutrophils (Nabe et al. 2011). Thus, while sharing OVA as a common allergen base, the choice of adjuvant shapes distinct mechanistic emphases and phenotypic details across these models (Fig. 2, Table 1).

#### Environmental exposure-induced models

Environmental exposure-induced models highlight the contribution of external stimuli to NA pathogenesis, among which HDM serves as the foundational stimulus and is widely used in modeling allergic airway diseases due to its clinical relevance (Kwak et al. 2021; Tage et al. 2024; Karaguzel et al. 2026). HDM, dominated by Dermatophagoides species, is a heterogeneous immunological mixture comprising allergenic proteases, Niemann-Pick type C2 family proteins, chitin, and microbial products such as LPS (Miller 2019). The proteases cleave epithelial tight junctions and activate protease activated receptor 2 on AECs, triggering alarmin release (Wan et al. 1999). Concurrent TLR activation promotes Th17 differentiation, leading to IL‑17A‑driven neutrophil recruitment, NLRP3 inflammasome activation, and IL‑1β release. The composition of different HDM extracts varies substantially, which may affect modeling outcomes (Post et al. 2012). Besides, different HDM exposure protocols induce distinct inflammatory phenotypes. Early-life repeated HDM exposure in BALB/c mice tends to generate an eosinophilic/T2-high phenotype characterized by increased IL-5, IL-13, immunoglobulin E (Puttur et al. 2026; Stölting et al. 2026). Short-term HDM exposure in adult C57BL/6 mice may induce mixed or T2-low airway inflammation (Tynecka et al. 2026). In contrast, prolonged high-dose HDM exposure shifts the response toward a neutrophilic phenotype suitable for NA studies (Hagner et al. 2020). For NA, total modeling periods range from 2 to 24 weeks: short‑term (2‑6 weeks) for acute neutrophilic inflammation, and chronic (8‑24 weeks) for remodeling and steroid resistance (Table 2). Other environmental triggers, such as cigarette smoke (CS) (Li et al. 2020; Hao et al. 2015; Tao et al. 2025; Belvisi et al. 2018; Yasuda et al. 2020) or diesel exhaust particles (Jung et al. 2021; Volder et al. 2023), have also been used in NA research, but they are often combined with HDM. The following mechanistic description focuses on HDM, which serves as the core stimulus.

**Table 2:** Characteristics of NA mouse models induced by environmental insults

Mouse	Sensitization		Challenge		Duration	Subset	Related mechanism	References
Strain (Sex, Age)	Agents (Routes)	Frequency	Agents (Routes)	Frequency				
BALB/c (F, 7–8 weeks)	25 μg HDM + 10 μg LPS (i.n.)	Day 0, 1, 2	25 μg HDM (i.n.)	Day 7–15	15 days	TH17, NEU	IL-24/IL-17A/STAT3/ERK pathway	Feng et al. 2022)
BALB/c (F, 8–10 weeks)	10 μg LPS + 100 μg HDM (i.n.)	Day 0, 1, 2, 7	50 μg HDM (i.n.)	Day 14, 15, 18, 19	20 days	NEU	IL-17 pathway	Kwak et al. 2021)
BALB/c (F, 6–8 weeks)	100 μg HDM extract (i.t.)	Day 0, 4	100 μg HDM extract (i.t.)	Day 14–17	17 days	TH1, TH17, NEU	Th17 pathway	Namakanova et al. 2022)
BALB/c (F, 8–10 weeks)	10 μg LPS + 100 μg HDM (i.n.)	Day 0, 1, 2, 7	50 μg HDM (i.n.)	Day 14, 15, 18, 19	20 days	NEU	NLRP3/IL-1β pathway; GRβ expression	Khalil et al. 2025)
BALB/c (F, 6 weeks)	50 μg OVA + 20 mg Alum (i.p.)	Day 0, 7, 14, 21	3% OVA (NEB) + CS (WBI)	Day 22–77	78 days	TH17, NEU, DC	TSLP/IL-23/TH17 pathway	Li et al. 2020)
BALB/c (F, 6 weeks)	20 μg OVA + 2 mg Alum (i.p.)	Day 0, 14	1% OVA (NEB) + CS (WBI)	Day 21–45	45 days	NEU	Steroid resistance	Hao et al. 2015)
BALB/c (F, 6–7 weeks)	50 μg OVA or 2 mg Alum (i.p.)	Day 0, 7, 14, 21	3% OVA (NEB) + CS (WBI)	Day 28–70	70 days	TH17, NEU	HDAC2/TH17/IL-17 pathway	Li et al. 2021)
BALB/c (F, 6 weeks)	100 μg HDM + 2 mg Alum (i.p.)	Day 0, 7	10 μg HDM extract (i.n.); 100 μg DEP (i.n.)	Day 14–20; Day 11–20	21 days	NEU	IL-17A pathway	Jung et al. 2021)
C57BL/6 (M/F, 6–8 weeks)	25 μg HDM + 10 μg LPS (OA)	Day 0, 1, 2	25 μg HDM (OA)	Day 7–14	15 days	NEU	IL-33/NETosis	Ma et al. 2021)
C57BL/6 (F, 8 weeks)	100 μg HDM + 100 μg CFA (s.c.)	Day 0	100 μg HDM (i.n.)	Day 14	14 days	NEU, Mφ	MIF/annexin-A1	Allam et al. 2023)
C57BL/6 (M, 3 months)	10 μg HDM (o.t.)	Day 0	10 μg HDM (o.t.)	Day 6–10	14 days	NEU, Mφ	NLRP3/TGF-β pathway; TNFα/CXCL1 pathway	Tage et al. 2024)
C57BL/6 (F, 6–7 weeks)	100 μg HDM + 100 μg OVA + 15 μg LPS + 2 mg Alum (i.p.)	Day 0, 1, 2	OVA (NEB); HDM (i.n.)	Day 14, 15, 18, 19	21 days	TH17, NEU	MBD2/TH17/IL-17 pathway	Duan et al. 2023)
C57BL/6 (F, 6–8 weeks)	20 μg HDM + 100 μL Alum (i.p.)	Day 0, 14	10 μg HDM (i.n.); CS (WBI)	3 times/week for 6 weeks; 5 days/week	9 weeks	TH17, DC	Autophagy/NETosis/TH17 pathway	Liu et al. 2025)
C57BL/6 (F, 6–8 weeks)	20 μg HDM + 66.7 μL Alum (i.p.)	Day 0, 14	10 μg HDM (i.n.); CS (WBI)	3 times/week for 5 weeks; 5 days/week	7 weeks	TH17, NEU, DC	IL-17/ferroptosis/NETosis	Tao et al. 2025)
C57BL/6 (M,/)	10 μg OVA + 100 μL Alum or 0.5 μg/kg HDM (i.p.)	Day 0, 14	2.5 mg/kg OVA or 1.25 μg/kg HDM (i.n.); CS (NEB)	Day24-26; Day 21–28	29 days	NEU	Steroid resistance	Belvisi et al. 2018)
C57BL/6 (F, 6–8 weeks)	1 μg HDM + 25 μg DEP (i.n.)	Day 1, 8, 15, 22, 29, 36	-	-	6 weeks	NEU, EOS	CXCL1/CXCL8/NETosis	Volder et al. 2023)

Representative HDM-based NA mouse models induced by CS, diesel exhaust particles, or combined protocols. Parameters include mouse strain, age, sex, sensitization/challenge protocols, duration, inflammatory subsets, mechanisms, and references, to aid protocol comparison and model selection

*Abbreviation*: *CS* cigarette smoke, *CXCL* C-X-C motif chemokine ligand, *DC* dendritic cell, *DEP* diesel exhaust particles, *EOS* eosinophil, *ERK* extracellular regulated protein kinases, *GRβ* glucocorticoid receptor β, *HDM* house dust mite, *IL* interleukin, *LPS* lipopolysaccharide, *MBD2* methyl-CpG binding domain protein 2, *MIF* migration inhibitory factor, *Mφ* macrophage, *NEB* nebulization, *NETosis* neutrophil extracellular trap formation, *NF-κB* nuclear factor kappa-B, *NEU* neutrophil, *NLRP3* NOD-like receptor pyrin domain-containing protein 3, *OA* oropharyngeal administration, *OVA* ovalbumin, *i.n.* intranasal, *i.t.* intratracheal, *o.t.* oral administration, *s.c.* subcutaneous, *STAT* signal transducer and activator of transcription, *TGF-β* transforming growth factor-β, *TH1* T helper cell 1, *TH17* T helper cell 17, *TSLP* thymic stromal lymphopoietin, *WBI* whole-body inhalation, *M* male, *F* female

**Fig. 3:**
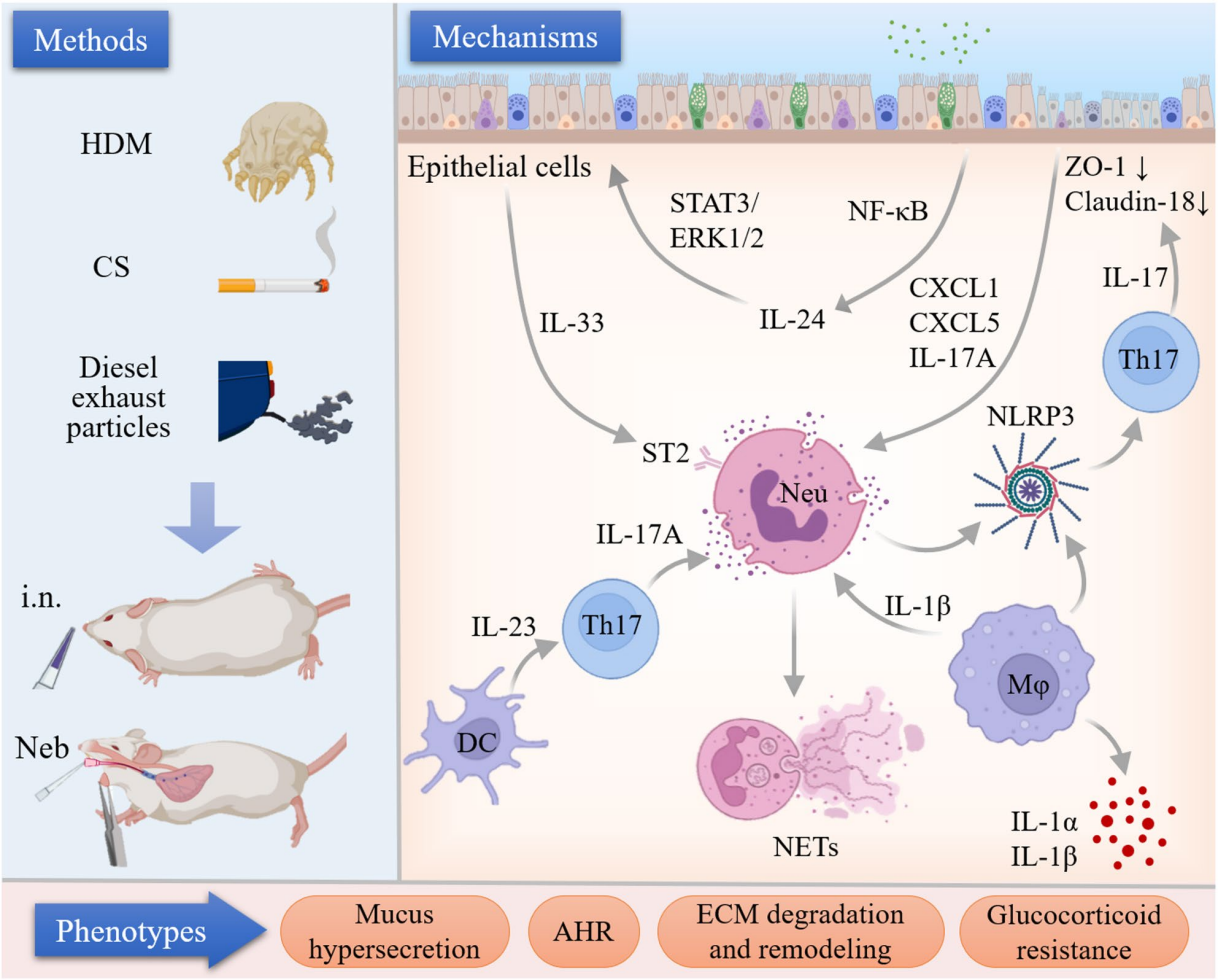
Methods and mechanisms of environmental exposure-induced NA mouse models. Environmental models employ clinically relevant stimuli, primarily HDM, CS and diesel exhaust particles, to replicate natural airway injury and inflammation. These exposures compromise epithelial barrier integrity, induce oxidative stress, activate innate immune signaling, and trigger alarmin and cytokine release. Abbreviation: AHR: airway hyperresponsiveness; CS: cigarette smoke; CXCL: C-X-C motif chemokine ligand; DC: dendritic cell; ECM: extracellular matrix; ERK1/2: extracellular signal-regulated kinase 1/2; HDM: house dust mite; IL: interleukin; Mφ: macrophage; MBD2: methyl-CpG-binding domain protein 2; Neu: neutrophil; NETs: neutrophil extracellular traps; NF-κB: nuclear factor kappa-light-chain-enhancer of activated B cells; STAT3: signal transducer and activator of transcription 3; ST2: suppression of tumorigenicity 2; Th: T helper cell

Proteolytic HDM disrupts epithelial tight junctions (e.g., Occludin, Claudin‑1, Zonula occludens-1), compromising barrier integrity and triggering release of damage-associated molecular patterns and alarmins (e.g., IL-33, thymic stromal lymphopoietin) (Liu et al. 2025; Khalil et al. 2025; Curren et al. 2023; Ma et al. 2021). These signals activate antigen-presenting cells like DCs and macrophages, which secrete IL-6, transforming growth factor-β (TGF-β) and IL-23, promoting Th17 differentiation (Tao et al. 2025; Feng et al. 2022; Namakanova et al. 2022). Additionally, the NLRP3 inflammasome activation increases caspase-1 signaling and IL-1β/IL-18 release, which further drives Th17 responses (Tan et al. 2019; Radzikowska et al. 2023). IL-17A produced by Th17 cells induces epithelial CXCL1 and CXCL5, enhancing neutrophil recruitment and NETosis (Jung et al. 2021; Li et al. 2021). IL-1β itself can downregulate tight junction proteins, perpetuating barrier dysfunction (Tan et al. 2019; Messaoud-Nacer et al. 2025). Furthermore, the inflammatory environment triggers excess oxidative stress, which further reduces HDAC2 activity, impairs glucocorticoid responsiveness, and reinforces Th17 polarization (Hao et al. 2015; Duan et al. 2023). Key phenotypes of environmental NA models include marked BALF neutrophilia, elevated IL‑17A, CXCL2 and CXCL5 (Yasuda et al. 2020; Tynecka et al. 2024). Histopathological assessments show neutrophilic peribronchial/perivascular infiltration, subepithelial collagen deposition, and goblet cell hyperplasia (Li et al. 2020). Functionally, the models exhibit increased AHR to methacholine (Volder et al. 2023), and chronic or combined models often develop steroid resistance (Kawagoe et al. 2021; Allam et al. 2023). Compared to OVA-adjuvant models that offer precise control for mechanistic dissection, HDM models better mimic natural allergen exposure and are particularly suited for studying environment-driven NA pathogenesis (Fig. 3, Table 2).

#### Pathogen-induced models

Pathogen‑triggered models focus on the driving role of infections in NA, often incorporating viral or bacterial components to mimic clinical acute exacerbations or chronic disease progression. Different pathogen classes operate through distinct mechanisms and are therefore best described separately.

##### Viral‑induced models

Rhinovirus (RV) and respiratory syncytial virus (RSV) are common respiratory pathogens. RV causes the common cold and triggers asthma exacerbations; it models acute neutrophilic airway inflammation in NA studies (Agache et al. 2025; Wirz et al. 2022; Hossain et al. 2020). RSV, a leading cause of pediatric lower respiratory infections (Billard And Bont 2023; Nguyen et al. 2016), is associated with asthma development and used to study infection‑induced asthma onset and long‑term airway remodeling (Billard And Bont 2023). Both viruses are recognized by TLR3 on AECs and TLR7 on plasmacytoid DCs, triggering type I interferon responses (Groskreutz et al. 2006; Kim et al. 2019). Viral replication induces IL-33 and thymic stromal lymphopoietin release, which activate Th17 cells to produce IL-17A, thereby amplifying neutrophil chemotaxis via CXCL1 and CXCL8 (Rajput et al. 2018; Mahmutovic Persson et al. 2018). In OVA‑based viral exacerbation models, BALB/c or C57BL/6 mice are typically sensitized via intraperitoneal injection of OVA with Alum before viral challenge (Hossain et al. 2020; Nguyen et al. 2016); in HDM‑based protocols, mice are sensitized intranasally with HDM prior to EV‑D68 or RV‑A1B infection (Rajput et al. 2018). During the challenge phase, viruses are administered via intranasal inoculation or nebulization, typically at 5 × 10⁶ plaque-forming unit for RV and approximately 0.5 × 10⁶ plaque-forming unit for RSV, usually delivered once (Hossain et al. 2020; Nguyen et al. 2016).

**Fig. 4:**
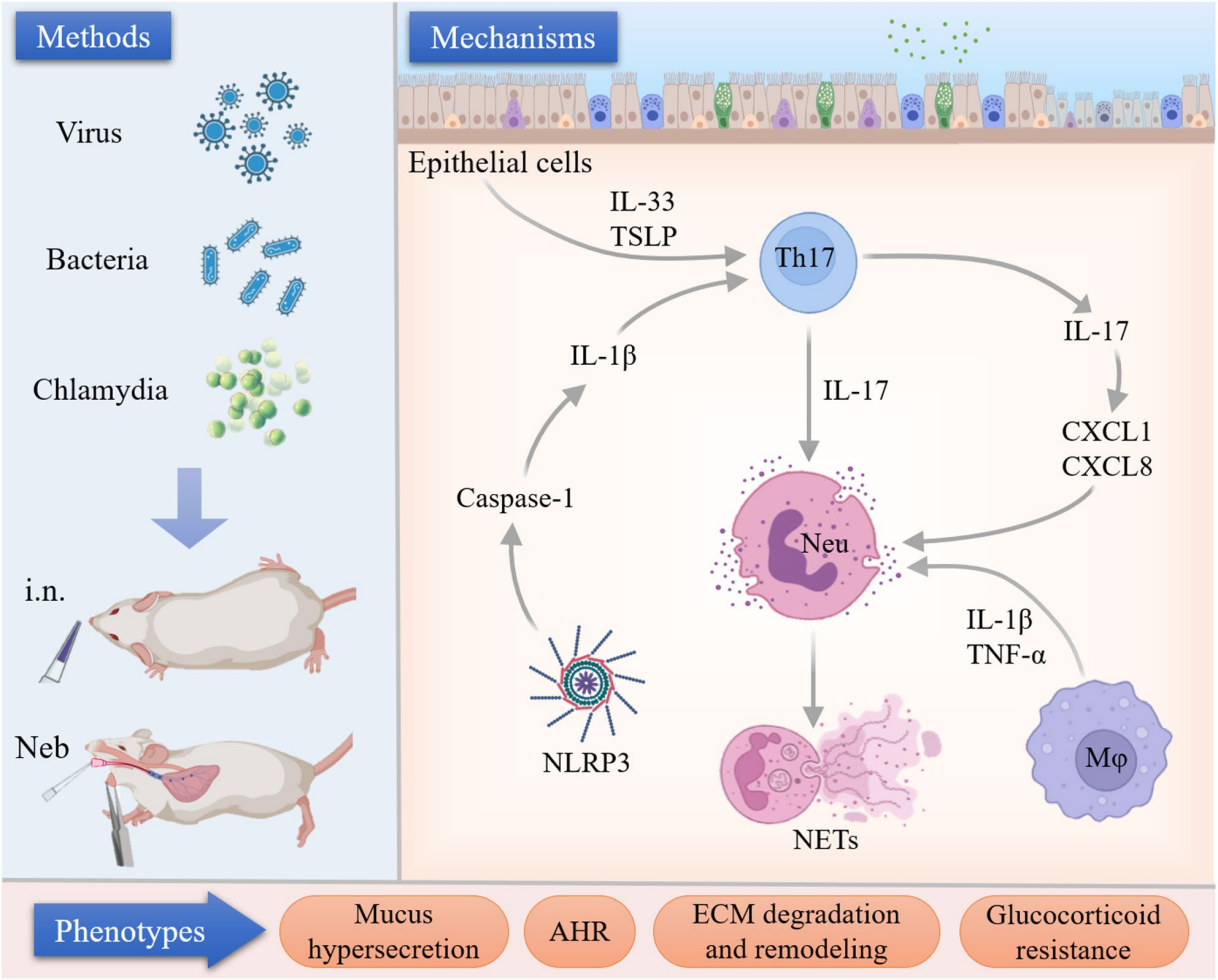
Methods and mechanisms of pathogen-induced NA mouse models. Pathogen‑triggered NA models mimic infection‑associated exacerbations and refractory inflammation. Viral stimuli induce alarmin release and Th17‑driven acute neutrophilia; bacterial stimuli activate TLRs/inflammasome pathways, sustaining IL‑1β/IL‑17‑mediated neutrophilic inflammation and steroid resistance. Abbreviation: AHR: airway hyperresponsiveness; CXCL: C-X-C motif chemokine ligand; ECM: extracellular matrix; IL: interleukin; Mφ: macrophage; Neu: neutrophil; NETs: neutrophil extracellular traps; Th17: T helper 17 cell; TSLP: thymic stromal lymphopoietin

Model duration is typically around 14 to 29 days for acute studies, although extended models may last up to 94 days (Curren et al. 2023; Hossain et al. 2020; Nguyen et al. 2016). Upon infection, viruses interact with AECs to induce the secretion of IL-33 and thymic stromal lymphopoietin (Mahmutovic Persson et al. 2018), activate antiviral innate immune pathways and promote IL-17-mediated neutrophilic inflammation (Hossain et al. 2020; Rajput et al. 2018). Viral infection also disrupts the Th17/regulatory T cell balance favoring Th17 polarization, leading to elevated IL-17A and amplified neutrophil recruitment and activation (Rajput et al. 2018). These viral and viral‑mimic exacerbation models recapitulate key acute NA features, including BALF and lung neutrophilia, elevated pro‑inflammatory mediators (e.g., IL‑17A, IL‑1β, TNF‑α, IL‑6, CXCL1), mucus‑related alterations, and enhanced AHR, with the specific phenotype depending on the protocol (Hossain et al. 2020; Nguyen et al. 2016; Rajput et al. 2018; Mahmutovic Persson et al. 2018) (Fig. 4, Table 3).

**Table 3:** Characteristics of NA mouse models induced by pathogens

Mouse	Sensitization		Challenge		Duration	Subset	Related mechanism	References
Strain (Sex, Age)	Agents (Routes)	Frequency	Agents (Routes)	Frequency				
BALB/c (M/F,/)	PVM; 1 μg CRE (i.n.)	Day 7, 42; Day 3, 45, 52, 59, 66	5 × 10⁶ TCID₅₀ RV1b (i.n.)	Day 94	94 days	NEU	IL-33/NETosis	Curren et al. 2023)
BALB/c (F, 8–10 weeks)	100 μg HDM extract (i.n.)	Day 0	10 μg HDM; 5 × 10^6^ ePFU EV-D68 or 5 × 10^6^ PFU RV-A1B (i.n.)	Day 11–12; Day 13	15 days	TH17, NEU, ILC3	IL-17 pathway	Rajput et al. 2018)
BALB/c (M, 5–6 weeks)	50 μg OVA + 1 mg Alum (i.p.)	Day 0	1% OVA (NEB); 0.5 × 10⁶ PFU RSV (i.n.)	Day 13–16; Day 19	24 days	TH17, NEU, Mφ	IL-17 pathway	Nguyen et al. 2016)
BALB/c (F, 6 weeks)	50 μg OVA (i.p.)	Day 0	2% OVA (NEB); NTHi OMVs (NEB)	Day 12–15; Day 10	16 days	TH17, NEU	IL-17 pathway	Zhang et al. 2023)
BALB/c (F, 6–8 weeks)	50 μg OVA (i.p.)	Day 0	10 μg OVA (i.n.); 5 × 10^5^ CFU NTHi (i.n.)	Day 12–15; Day 10	21 days	TH17, NEU	IL-17A pathway; TLR4 pathway	Essilfie et al. 2012)
BALB/c (F, 6–8 weeks)	100 μg OVA + 2 mg Alum (i.p.)	Day 0, 7, 14	5% OVA (NEB); 5 × 10^5^ CFU NTHi (NEB)	Day 21–24; Day 25	28 days	TH17, NEU	IL-17 pathway; MAPK pathway	Wang et al. 2019)
BALB/c (M, 6–8 weeks)	50 μg OVA + 1 mg Alum (i.p.)	Day 0	1% OVA (NEB); 2 × 10⁶ CFU NTHi (i.n.)	Day 13–16; Day 14	24 days	TH17, NEU, Mφ	ROS/NF-κB pathway; TH17/IL-17 pathway	Zhang et al. 2020)
BALB/c (M/F, 6–8 weeks)	50 μg OVA (i.p.)	Day 0	10 μg OVA (i.n.); 100 IFU C. muridarum or 2 × 10⁶ CFU H. influenzae (i.n.)	Day 12–13, 33–34; Day 14	35 days	NEU, Mφ	NLRP3/caspase-1/IL-1β pathway	Kim et al. 2017)
C57BL/6 (M, 8–10 weeks)	25 μg HDM extract (i.n.)	Every 2 days for first 3 weeks	100 μg Poly I:C (i.n.)	Day 21–23	24 days	TH1, NEU	IL-1β pathway	Mahmutovic Persson et al. 2018)
C57BL/6 (F, 8–12 weeks)	25 μg HDM (i.n.)	Day 0, 7	10 μg HDM + 60/200 μg Poly I:C (i.n.)	Day 14–16	17 days	TH1, TH17, NEU, Mφ	IL-6//NETosis/PANoptosis; STING/cGAS pathway	Messaoud-Nacer et al. 2025)
C57BL/6 (M/F, 6–8 weeks)	100 μg OVA + 2 mg Alum (i.p.)	Day 0	50 μg OVA; 1 × 10⁷ TCID₅₀ hRV 1B (i.n.)	Day 10–12; Day 12	14 days	TH17, NEU	TH17/IL-17A pathway	Hossain et al. 2020)

Representative pathogen-associated NA mouse models induced by viruses, bacteria, or combined protocols. Parameters include mouse strain, age, sex, sensitization/challenge protocols, duration, inflammatory subsets, mechanisms, and references, to aid protocol comparison and model selection

*Abbreviation*: *CFU* colony-forming unit, *C. muridarum* Chlamydia muridarum, *CRE* cockroach extract, *cGAS* cyclic GMP-AMP synthase, *CXCL* C-X-C motif chemokine ligand, *DC* dendritic cell, *EV-D68* enterovirus D68, *ePFU* egg plaque-forming unit, *HDM* house dust mite, *H. influenza* Haemophilus influenzae, *hRV 1B* human rhinovirus 1B, *IFU* infectious unit, *IL* interleukin, *Mφ* macrophage, *NETosis* neutrophil extracellular trap formation, *NEB* nebulization, *NEU* neutrophil, *NF-κB* nuclear factor kappa-B, *NLRP3* NOD-like receptor pyrin domain-containing protein 3, *NTHi* nontypeable Haemophilus influenzae, *OMVs* outer membrane vesicles, *Poly I:C* polyinosinic-polycytidylic acid, *PVM* pneumonia virus of mice, *ROS* reactive oxygen species, *RV1b* rhinovirus 1b, *RV-A1B* rhinovirus A1B, *RSV* respiratory syncytial virus, *STING* stimulator of interferon genes, *TCID₅₀* 50% tissue culture infective dose, *TH1* T helper cell 1, *TH17* T helper cell 17, *M* male, *F* female

##### Bacterial‑induced models

Non-typeable Haemophilus influenzae (NTHi) is an opportunistic upper respiratory commensal causing otitis media, sinusitis, and COPD exacerbations (Su et al. 2023; 2022). As a mucosal Gram-negative bacterium, NTHi activates innate immune pathways in AECs and macrophages, particularly TLR2 signaling, triggering NF-κB-dependent production of IL-1β and TNF-α, making it a common model for chronic bacterial infection and persistent airway inflammation in NA (Zhang et al. 2023; Xu et al. 2011). In contrast, Chlamydia muridarum is an obligate intracellular bacterium that causes pharyngitis, bronchitis, and pneumonia (Kocher et al. 2024). It activates the NLRP3 inflammasome, leading to IL-1β secretion, which drives an IL-17-associated neutrophilic inflammation with steroid resistance (Kim et al. 2017). Its ability to establish persistent infection and alter the airway immune microenvironment makes it useful for studying both acute infection-induced inflammation and long-term immune dysregulation in NA (Kim et al. 2017). Most bacterial-induced models use OVA-based sensitization, with or without Alum, followed by NTHi or Chlamydia respiratory infection (Kim et al. 2017; Zhang et al. 2023; Essilfie et al. 2012; Zhang et al. 2020). NTHi is given intratracheally or intranasally at 0.5 × 10^6^–2 × 10⁶ colony-forming unit, often with repeated OVA aerosol challenges (Essilfie et al. 2012; Zhang et al. 2020; Wang et al. 2019). Chlamydia muridarum is inoculated intranasally at around 100 infectious-forming unit, sometimes combined with OVA challenges (Kim et al. 2017). Model duration ranges from 16 to 35 days. Upon infection, bacteria activate Th17 responses via TLRs on AECs and immune cells, with Chlamydia additionally engaging the NLRP3 inflammasome, leading to elevated IL-17A, IL-1β, and neutrophil recruitment (Kim et al. 2017; Essilfie et al. 2012; Wang et al. 2019). These bacterial models recapitulate key NA features, including BALF and lung neutrophilia, elevated Th17/IL-17 and IL-1β responses, mucus hypersecretion, enhanced AHR, and reduced corticosteroid responsiveness under severe infection (Kim et al. 2017; Essilfie et al. 2012; Wang et al. 2019) (Fig. 4, Table 3).

Viral models are particularly valuable for simulating acute exacerbations and studying alarmin‑driven pathways. While bacterial models excel in representing chronic infection‑related refractory inflammation and long‑term, infection‑mediated pathological reprogramming of the airway. These pathogen-triggered models, together with the two aforementioned types, form a complementary toolkit that addresses diverse research needs. OVA-based systems allow high controllability and subtype-specific investigations, making them ideal for mechanistic validation and preliminary drug screening. Environmental models recapitulate natural disease initiation, while pathogen-driven approaches focus on infection-induced inflammation and treatment resistance. Collectively, these models enable targeted mechanistic inquiry, physiological scenario simulation, and infection-focused exploration, forming an integrated framework for comprehensive NA research.

#### Obesity-related models

Obesity‑associated NA models are highly clinically relevant, simulating NA under obese conditions via dietary or fatty acid interventions (Althoff et al. 2024). The HFD model reflects modern high‑calorie dietary patterns and enables study of metabolic dysfunction and its respiratory impact (Son et al. 2024; Yang et al. 2024); palmitate, a saturated fatty acid abundant in human diets, directly elicits lipid‑induced inflammation (Chen et al. 2024; Dimasuay et al. 2023). They can activate TLR4 on macrophages and AECs, triggering TNF-α, IL-6, and IL-1β secretion, which shift Th17/regulatory T cell balance and recruit neutrophils via CXCL1/CXCL5 (Yang et al. 2024; Mohamed And Amrani 2024). Additionally, obesity-upregulated leptin directly enhances neutrophil survival and activation (Wang et al. 2023).

Strain selection for the models is flexible, though 6–8-week-old mice are typically chosen to ensure metabolic naivety prior to intervention (Tashiro et al. 2017). Obesity and airway responses are induced by HFD or lipid administration, often with HDM challenge, over 4–10 weeks, with HDM applied during the final 1–2 weeks (Mohamed And Amrani 2024).

Mechanistically, excess lipids activate TLR4, which promote secretion of IL-17A, TNF-α, IL-1β, macrophage inflammatory protein 2, and CXCL1, driving neutrophil recruitment and activation (Mohamed And Amrani 2024; Wang et al. 2023). Metabolic imbalance skews immune polarization toward pro-inflammatory Th17 cells and away from regulatory T cells, further amplifying neutrophilic inflammation (Tashiro et al. 2017). These TLR4-dependent, non-Th2 pathways align with clinical observations that obesity shifts asthma toward a neutrophilic phenotype. Key NA features recapitulated include: increased BALF neutrophils, elevated non-Th2 cytokines/chemokines in BALF and lung tissue (Tashiro et al. 2017), severe neutrophilic infiltration around airways and blood vessels (Mohamed And Amrani 2024), goblet cell hyperplasia with mucus overproduction detectable via PAS staining, and early airway remodeling (e.g., collagen deposition) in chronic models (Wang et al. 2023). Marked methacholine‑induced AHR reflects compromised lung function in NA. Thus, HFD- or palmitate-induced models are highly clinically relevant, driven by metabolic dysfunction and non‑Th2 signaling, and closely mirror human obesity‑related NA (Table 4).

**Table 4:** Characteristics of NA mouse models with other induction types

Mouse	Special type	Sensitization		Challenge		Duration	Subset	Related mechanism	References
Strain (Sex, Age)		Agents (Routes)	Frequency	Agents (Routes)	Frequency				
BALB/c (F, 3 weeks)	HFD: 60% calories from fat for 10 weeks	25 μg HDM extract (i.n.)	once weekly for 3 times after 7-week HFD	5 μg HDM extract (i.n.)	3 consecutive days after 10-week HFD	74 days	TH1, NEU, Mφ	SFA/IL-1β pathway	Tashiro et al. 2017)
BALB/c (F, 6 weeks)	PA administration: 50 μM, i.p., 5 times/week for 4 weeks	25 μg HDM extract (i.n.)	Day 2, 9, 16	5 μg HDM extract (i.n.)	Day 23–25	4 weeks	TH1, NEU, Mφ	SFA/IL-1β↑ pathway	Tashiro et al. 2017)
C57BL/6 (M/F, 14–15 weeks)	HFD: 42% calories from fat for 10 weeks	25 μg HDM extract (i.n.)	Day 1 after 7-week HFD	25 μg HDM extract (i.n.)	Day 4, 6, 11, 13, 19 after sensitization	10 weeks	TH17, NEU, Mφ	IL-17 pathway; IL-1β pathway	Mohamed And Amrani 2024)
C57BL/6 (F, 6–8 weeks)	HFD: 60% calories from fat for 16 weeks	0.1 μg LPS + 100 μg OVA (i.t.)	Day 0, 7 after 16-week HFD	5% OVA (NEB)	Day 14 after sensitization	18 weeks	NEU, Mφ	JNK/STAT3/AKT/CXCL2 pathway; M1 polarization	Wang et al. 2023)
BALB/c (M, 8–10 weeks)	Four consecutive days of exposure	-	-	2 μg LPS (i.n.)	Day 1–4	4 days	ILC3, NEU, DC	IL-1β pathway; IL-17 pathway	Jonckheere et al. 2022)
BALB/c (F, 8–12 weeks)	DRA exposure	100 μL DRA (5 μg dust mite + 50 μg ragweed + 5 μg Aspergillus + Alum) (s.c.)	Day 0, 7	15 μL DRA (i.n.)	2 days/week for week 3–10	10 weeks	NEU, Mφ	NF-κB/IL-8 pathway	Liu et al. 2013)
C57BL/6 (F, 6 weeks)	Dog allergen exposure	10 IR dog allergen (i.n.)	Day 1–5	10 IR dog allergen (i.n.)	5 days/week for week 3–5	33 days	TH17, NEU	TH17/IL-17A pathway	Margelidon-Cozzolino et al. 2025)

Representative NA mouse models induced by high‑fat diet, low‑dose LPS, dog allergen, or multi‑allergen. Parameters include mouse strain, age, sex, sensitization/challenge protocols, duration, inflammatory subsets, mechanisms, and references, to aid protocol comparison and model selection

*Abbreviation*: *AKT* Protein Kinase B, *CXCL C-X-C* motif chemokine ligand, *DC* dendritic cell, *DRA* dust mite, ragweed, and Aspergillus species, *HFD* high-fat diet, *HDM* house dust mite, *IL* interleukin, *ILC3* type 3 innate lymphoid cell, *IR* international unit, *i.n.* intranasal, *i.t.* intratracheal, *JNK* c-Jun N-terminal kinase, *LPS* lipopolysaccharide, *Mφ* macrophage, *NEB* nebulization, *NEU* neutrophil, *NF-κB* nuclear factor kappa-B, *OVA* ovalbumin, *PA* palmitic acid, *SFA* saturated fatty acid, *s.c.* subcutaneous, *STAT3* signal transducer and activator of transcription 3, *TH1* T helper cell 1, *TH17* T helper cell 17, *M* male, *F* female

#### Other murine models

In addition to mainstream NA mouse models, several alternative approaches have been developed to mimic rare or distinct clinical forms. Here, we summarize three representative models. The canine allergen-induced mouse model of NA is valuable for studying T2-low severe asthma. It typically uses 6-week-old female C57BL/6 J mice sensitized intranasally with canine allergen for 5 consecutive days, followed by 3 weeks of challenge (5 times per week, approximately 33 days total). Mechanistically, IL-17A pathway activation drives neutrophilic inflammation and steroid resistance. Phenotypes include BALF neutrophilia, high IL-17A level, and features of airway remodeling such as mucus hypersecretion, subepithelial fibrosis, and smooth muscle hypertrophy. It recapitulates steroid-resistant T2-low severe asthma, but its single allergen and relatively moderate duration limit its use in high‑throughput studies and the investigation of more complex or chronic disease features (Margelidon-Cozzolino et al. 2025).

The low-dose LPS-induced NA model applies 8–10-week-old male BALB/c mice. Modeling is achieved via intranasal administration of 2 μg LPS for 4 consecutive days, resulting in a short cycle of only 4 days. Mechanistically, epithelial IL-1β activates group 3 innate lymphoid cells to secrete IL-17A and IL-22, driving neutrophilic inflammation and AHR. Phenotypically, it shows BALF neutrophilia, elevated IL-1β and IL-17A, and significant AHR. Its advantages include rapid construction and suitability for acute, non-allergic mechanistic studies, but it does not capture chronic airway remodeling and is less relevant for allergen-driven NA (Jonckheere et al. 2022).

The multi-allergen-induced NA model uses 8–12-week-old female BALB/c mice. A mixed allergen (DRA: dust mite, ragweed, aspergillus) supplemented with 0.4 mg/mL LPS serves as the inducer. The protocol involves subcutaneous sensitization on days 0 and 7, followed by twice-weekly nebulization with 1% DRA from day 21 to 56, totaling 8 weeks. Mechanistically, mite proteases activate NF-κB to induce epithelial IL-8, driving neutrophil infiltration and steroid resistance. Phenotypes include high BALF neutrophil proportion, elevated IL-8 and TGF-β, and complete glucocorticoid unresponsiveness. It excels at simulating chronic asthma with complex environmental triggers, but the long duration and complex allergen mix hinder mechanistic dissection and reproducibility (Liu et al. 2013). These three models encompass distinct scenarios, enriching the NA model system; researchers can select an appropriate model accordingly (Table 4).

### Rat models

Rat NA models, commonly using Wistar, PVG, or Sprague–Dawley strains, enable flexible recapitulation of NA features and offer particular advantages for studying corticosteroid resistance (Qingwu et al. 2014). OVA serves as the core sensitizing antigen, often combined with adjuvants such as Freund’s adjuvant, Alum, or aluminum hydroxide. Sensitization is typically performed via 1–2 subcutaneous or intraperitoneal injections, with protocols adjusted based on the desired model duration (Brochetti et al. 2022). The challenge phase generally involves OVA aerosol inhalation; some protocols incorporate viral infection to mimic asthma exacerbation. Challenge frequency and duration vary, yielding total model timelines ranging from 23 to 56 days (Qingwu et al. 2014; Lauzon-Joset et al. 2018).

Key metrics include elevated BALF neutrophils, MPO activity, pro-inflammatory cytokines such as IL-17, IL-6, TNF-α, and sometimes leukotriene B4 or high-mobility group box 1, along with reduced anti-inflammatory IL-10 (Qingwu et al. 2014). Histopathological assessment reveals inflammatory cell infiltration, mast cell degranulation, goblet cell hyperplasia, collagen deposition, and α-smooth muscle actin upregulation. In steroid-resistant models, dexamethasone fails to suppress inflammatory cell migration, cytokine overproduction or AHR (Brochetti et al. 2022). Strengths of the models are reliability, high reproducibility, and suitability for mechanistic and pharmacologic studies. Limitations include incomplete coverage of NA immune endotypes by any single protocol, and species-specific differences that may hinder clinical translatability (Table 5).

**Table 5:** Characteristics of rat and equine models of NA

	Animals	Sensitization		Challenge		Duration	Subset	Related mechanism	References
	Strain (Sex, Age)	Agents (Routes)	Frequency	Agents (Routes)	Frequency				
Rat	Wistar (M,/)	20 μg OVA + 75 μL FA (s.c.)	Early stage	1% OVA (NEB)	Day 21 and 22	23 days	TH17, NEU	IL-17 pathway	Brochetti et al. 2022)
	PVG (M, 8–12 weeks)	500 μL OVA/Alum mixture (i.p.)	14 days before challenge	1% OVA (NEB); 10⁷ PFU vMC₀ (i.n.)	Day 1 post-viral infection; Day 0	16 days	TH17, NEU, Mφ	M2 polarization/TH17 pathway	Lauzon-Joset et al. 2018)
	Sprague Dawley (M, 7–8 weeks)	100 mg OVA + 100 mg Alum + 6 × 10⁹ Bordetella pertussis (i.p.); 10 μg LPS (i.t.)	Day 0 and 7; Day 0, 7, 21, 35	1%−11%, wt/vol OVA (NEB)	5 days/week for 6 weeks (starting from Day 14)	56 days	TH17, NEU	HMGB1/IL-17 pathway	Qingwu et al. 2014)
Equine	Not specified (M/F, 12–16 years)	-	-	Organic dust (Natural inhalation)	Long-term exposure	5-month intervention	NEU, Mφ	IL-1β pathway	Mainguy-Seers et al. 2022)
	Warmblood horses (M/F, Adult)	-	-	Organic dust (Natural inhalation)	Long-term exposure	Chronic, lifelong	TH17, NEU	IL-17 pathway;	Simoes et al. 2022)
	Warmblood horses (F/gelding, 7–19 years)	-	-	Organic/inorganic dust (Natural inhalation)	Long-term exposure	Chronic, lifelong	TH17, NEU, DC, Mφ	Th17/IL-17 pathway; CXCL13/ B cell pathway	Sage et al. 2024)
	Warmblood horses (M/F, ≥ 7 years adult)	-	-	Organic dust (Natural inhalation)	Long-term exposure	Chronic, lifelong	NEU, Mφ	TGF-β pathway	Bullone And Lavoie 2020)

Representative NA rat and equine models. Parameters include animal strain, age, sex, sensitization/challenge protocols, duration, inflammatory subsets, mechanisms, and references, to aid protocol comparison and model selection

*Abbreviation*: *CXCL* C-X-C motif chemokine ligand, *DC* dendritic cell, *FA* Freund's adjuvant, *HMGB1* high mobility group box 1 protein, *i.n.* intranasal, *i.p.* intraperitoneal, *i.t.* intratracheal, *s.c.* subcutaneous, *INH* inhalation, *IL* interleukin, *LPS* lipopolysaccharide, *Mφ* macrophage, *NEB* nebulization, *NEU* neutrophil, *OVA* ovalbumin, *TGF-β* transforming growth factor-β, *TH17* T helper cell 17, *M* male, *F* female

### Equine models

Equine models develop NA spontaneously, allowing long‑term observation of disease progression without artificial induction (Sheats et al. 2019). Most studies use adult horses aged ≥ 7 years (median age around 12 years), including Warmbloods, Quarter Horses, Thoroughbreds, with no gender restriction (mares, stallions, geldings) (Mainguy-Seers et al. 2022; Lange-Consiglio et al. 2019). Disease arises from natural inhalation of organic dusts containing fungal spores, endotoxins, and β‑D‑glucan. Some protocols enhance phenotypes through hay dust suspensions (10 mg/mL), LPS (10–50 μg/horse), or aspergillus extract (10⁶ colony-forming unit/mL) (Hunter et al. 2020; Conley et al. 2025). Spontaneous models rely on long-term environmental exposure, while experimental challenge frequency can be adjusted as needed (Sage et al. 2024). Disease progression is chronic, often lasting years naturally, though experimental interventions range from hours to 5 months (Bullone And Lavoie 2020).

Mechanistically, equine NA involves IL-17/NF-κB pathway activation, Th17-driven neutrophil recruitment, NETosis, and TLR4/MyD88 signaling (Simoes et al. 2022). Other studies implicate the Myristoylated Alanine Rich C Kinase Substrate protein in modulating reactive oxygen species production, and microRNAs (miR-128, miR-744) in regulating neutrophil activity (Conley et al. 2025; Simoes et al. 2022). Moreover, monocyte-T cell interactions promote Th17 polarization, amplifying neutrophilic inflammation (Davis and Sheats 2021). Phenotypically, BALF reveals markedly increased neutrophils (Bullone And Lavoie 2020). Histopathology exhibits airway remodeling (smooth muscle hyperplasia, goblet cell metaplasia/hyperplasia, peribronchial collagen deposition, and mucus plugging), and AHR is confirmed via methacholine or histamine challenge (Hunter et al. 2020). Collectively, these models closely mimic human occupational or seasonal NA from organic dust. Equine airway anatomy resembles humans, permitting repeated BALF sampling, lung biopsies, and long-term imaging (Simoes et al. 2022). Main limitations include high operational costs, need for veterinary expertise, genetic variability, and constraints from natural disease course or seasonality (Sheats et al. 2019; Davis and Sheats 2021) (Table 5).

## In vitro models

### Air–liquid interface (ALI) models

The ALI culture technique has become a cornerstone for constructing in vitro models of asthma, as it closely mimics the in vivo physiological microenvironment of the airway epithelium, with the apical surface exposed to air and the basolateral side immersed in nutrient medium (Pezzulo et al. 2011). This configuration faithfully recapitulates the pseudostratified structure and functional polarity of the native airway epithelium (Baldassi et al. 2021). Primary human bronchial or tracheal epithelial cells, isolated from asthmatic patients via brushing or biopsy, are preferred as seed cells because they retain disease-specific genetic backgrounds and NA-related pathological traits (Valdez et al. 2024; Upadhyay et al. 2024; Allard et al. 2019). Immortalized cell lines may also be used for standardized assays. Specifically, cells are first expanded in submerged culture to form a confluent monolayer. Upon switching to ALI conditions, they undergo air‑induced differentiation over 2‑4 weeks, developing into a polarized, pseudostratified epithelium that comprises multiple cell types: basal cells, club cells, ciliated cells, and goblet cells (Baldassi et al. 2021; Lamb et al. 2021; Jackson et al. 2020). This differentiated epithelium exhibits high transepithelial electrical resistance and tight junction integrity, making it suitable for barrier function studies (Wang et al. 2019). ALI cultures can be assessed for asthma‑relevant endpoints, including epithelial barrier integrity, mucus production, epithelial thickening, and chemokine release (Keeler et al. 2023; Gao et al. 2022; Castaneda et al. 2023).

**Fig. 5:**
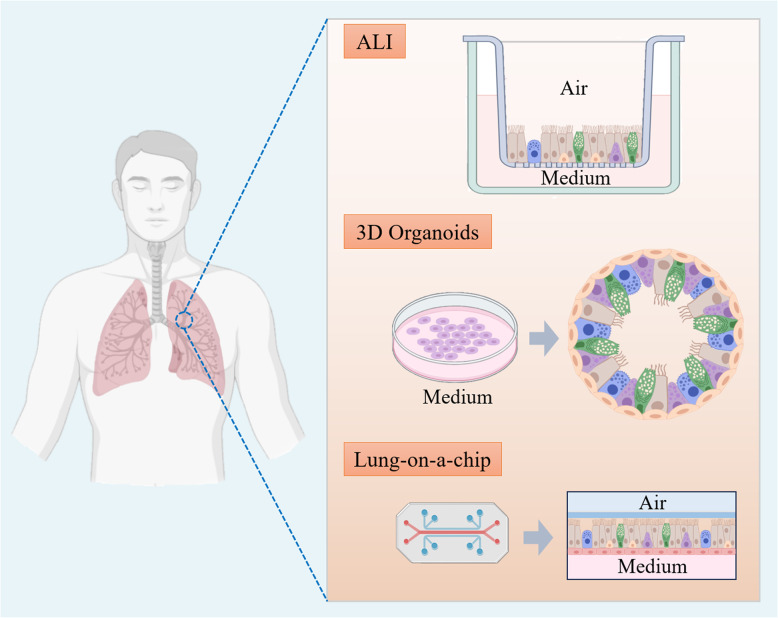
In vitro models of NA. Advanced in vitro platforms, including ALI cultures, airway organoids, and lung-on-a-chip systems. Abbreviation: ALI: air–liquid interface

To better simulate the airway microenvironment in NA, ALI cultures can be extended into multicellular co-culture systems (Jevnikar et al. 2019). For example, bronchial smooth muscle cells from asthmatic donors can be cultured basolaterally to investigate CCL5-mediated epithelial inflammation (Allard et al. 2019). Alternatively, differentiated THP-1 macrophages, primary monocyte-derived macrophages, or LAD2 mast cells may be introduced on either side of the porous membrane to model epithelium-immune crosstalk (Murphy et al. 2023). When co‑cultured with primary human neutrophils, these systems further allow assessment of neutrophil recruitment and activation, which correlate with epithelial dysfunction, mucus overproduction, and impaired steroid responsiveness, all central features of severe NA. Through precise cell sourcing, standardized differentiation, specific inflammatory stimulation, and reduced structural complexity, ALI culture reliably models core NA features such as epithelial barrier dysfunction, neutrophilic inflammation, and mucus hypersecretion. It serves not only as a stand‑alone platform but also as a fundamental unit in organoid and lung‑on‑a‑chip systems, enhancing physiological relevance and complementing in vivo models (Fig. 5).

### Organoid models

Organoid models, derived from primary cells or stem cells, recapitulate airway epithelial pathology in three-dimensional structures, capturing patient-specific NA features (Gras et al. 2012; Zhou et al. 2023). Primary human bronchial epithelial cells from asthmatic patients differentiate under ALI conditions into pseudostratified epithelium (basal, ciliated, and goblet cells), modeling mucociliary phenotypes (Petpiroon et al. 2023). Airway basal stem cells from bronchial brushings can self‑assemble into self‑renewing three-dimensional organoids that preserve donor‑specific genetic and epigenetic traits (Kuhl et al. 2023). Disease‑relevant phenotypes are induced by cytokines: IL-13 promotes goblet cell metaplasia via NOTCH2 signaling (Barkauskas et al. 2017), while TGF-β triggers epithelial-mesenchymal transition with α-smooth muscle actin expression (Dichtl et al. 2024). Environmental stimuli like particulate matter and engineered nanoparticles further exacerbate inflammation and IL-8 release (Petpiroon et al. 2023; Aydin et al. 2021).

Phenotypically, severe asthma organoids show thicker reticular basement membrane, increased epithelial thickness, higher goblet cell proportion, apical mucus granules, and preserved cilia compared to mild asthma models (Zscheppang et al. 2018). Exposure to NA-associated cytokines and neutrophil-derived factors induces chemokine release, epithelial barrier disruption, mucus overproduction, and neutrophil-mediated injury. These platforms also assess steroid resistance and remodeling responses. For drug screening, they recreate the pro‑inflammatory/anti‑inflammatory imbalance of severe disease, enabling evaluation of therapies targeting neutrophil recruitment or mucus regulation. For personalized medicine, patient-derived organoids retain severity-specific features and can predict individual therapeutic outcomes. Limitations include lack of integrated immune or vascular components and insufficient protocol standardization. Overall, by preserving inherent pathological differences and supporting three-dimensional dynamic culture, airway organoids effectively replicate mucus obstruction, inflammatory imbalance, and structural remodeling, offering a physiologically relevant platform for NA mechanistic studies and drug screening (Fig. 5).

### Lung-on-a-chip models

Lung-on-a-chip devices utilize microfluidic systems to simulate respiratory dynamics, integrating ALI culture and multicellular components to mimic tissue‑level NA pathology (Kim et al. 2023). They typically employ polydimethylsiloxane or polyetheretherketone substrates with ALI zones, co-culture chambers, and dynamic control modules (Min et al. 2025; Zhu et al. 2022). ALI is established on porous membranes, with AECs cultured on the apical side and vascular endothelial or stromal cells basolaterally (Barros et al. 2021). Some devices incorporate elastic membranes and pumps to simulate breathing motions and control fluid flow or rhythmic stretch (Liu et al. 2025; Bovard et al. 2018). Asthma modeling involves multidimensional regulation. Cellular components often include Calu-3 cells or primary human bronchial epithelial cells co-cultured with pulmonary microvascular endothelial cells (Nawroth et al. 2020). Pathological features are induced by stimuli such as basolateral TNF-α, triggering mucus hypersecretion and IL-8 release (Monteduro et al. 2023). Human rhinovirus 16 infection simulates viral exacerbation, disrupting the secretion timing of interferon-λ1 and IL-6, and promoting neutrophil transendothelial migration (Nawroth et al. 2020). Crucially, introduction of human neutrophils under physiological shear stress models adhesion, transmigration, activation, and NET-associated epithelial damage, coupled with barrier dysfunction and mucus overproduction. Glucocorticoids fail to suppress these integrated responses, underscoring the platform's utility in recapitulating steroid-resistant, neutrophil-driven asthma pathology. These systems successfully model neutrophil transmigration, inflammatory cytokine release, and mucus hypersecretion. They are particularly useful for evaluating inhaled therapies and, when using patient-derived primary cells, for predicting individualized therapeutic responses. However, their widespread application is currently tempered by high fabrication costs and limited experimental throughput. Overall, these advanced platforms offer a highly physiologically relevant tool for mechanistic and therapeutic studies of NA (Fig. 5).

## Conclusion and outlook

This review has systematically evaluated and integrated findings from both in vivo and in vitro models of NA, uncovering consistent pathophysiological mechanisms and highlighting challenges that hinder translational progress. Clinically, NA is characterized by sustained neutrophilic predominance in induced sputum (with substantial day-to-day variability but persistent elevation in severe cases), late-onset severe disease, comorbidities such as smoking and obesity, poor glucocorticoid responsiveness, and frequent exacerbations induced by infections or environmental irritants rather than allergens. Pathophysiologically, it is defined by Th1/Th17-dominant inflammation driven by IL-17A, NETosis, primary epithelial barrier dysfunction that actively promotes inflammation, and specific molecular mechanisms of steroid resistance (e.g., HDAC2 inactivation). These unique features distinguish NA from other asthma endotypes, establish a clearer conceptual framework, and facilitate the development of targeted therapies for the disease.

A key takeaway is that no single model captures the full spectrum of human NA; instead, researchers should align their choice of model with the specific research question at hand. Specifically, for dissecting Th17‑driven neutrophil recruitment and signaling, OVA combined with CFA or LPS in mice offers a well‑controlled, high‑throughput system that robustly elevates IL‑17A, CXCL1/CXCL5, and airway neutrophilia, making it suitable for testing anti‑IL‑17 or CXCR2 antagonists. When the primary interest is steroid resistance or screening steroid‑restoring therapies, rat models and multi‑allergen mouse models reliably reproduce glucocorticoid hyporesponsiveness, often via HDAC2 downregulation. To study chronic airway remodeling and long‑term disease progression, the equine model, which develops NA spontaneously over years, uniquely recapitulates smooth muscle hypertrophy, peribronchial fibrosis, and mucus plugging, and is ideal for testing anti‑remodeling agents despite higher cost and lower throughput. Besides, for environmental or pollution‑driven NA, HDM combined with CS or diesel exhaust particles in chronic exposure protocols captures the complexity of real‑world triggers and is recommended for studying oxidative stress‑induced steroid resistance or epithelial alarmin activation. To investigate infection‑associated acute exacerbations, viral models are best suited for short‑term studies of epithelial alarmin release and NETosis, whereas bacterial models better represent chronic, therapy‑refractory inflammation and NLRP3‑dependent mechanisms. For human‑specific, mechanism‑focused studies, ALI cultures, patient‑derived organoids, and lung‑on‑a‑chip devices allow precise dissection of epithelial barrier dysfunction, neutrophil transmigration, and personalized drug testing in a human genetic background, particularly valuable for validating findings from animal models and screening inhaled therapeutics. Here, we provide a concise overview of murine NA models in Table 6, summarizing their induction methods, distinctive advantages, and mechanistic bases to facilitate rational model selection.

**Table 6:** Comparisons of features of NA mouse models with different induction types

		OVA with adjuvants	HDM	CS	Virus	Bacteria	Obesity-related
Immune cells	TH1	+ +	+	+	+	+	+ +
	TH17	+ + + +	+ +	+ + +	+ + +	+ + +	+
	NEU	+ + + +	+ + + +	+ + + +	+ + + +	+ + + +	+ + + +
	Mφ	+ +	+	+	+ +	+	+ + + +
	DC	+	NR	+ +	NR	NR	NR
	EOS	NR	+	NR	NR	NR	NR
Molecules	IL-1	+ +	+	+	+	+	+ +
	IL-17	+ + + +	+ +	+ + +	+ + +	+ + +	+
	IL-6	+	+	NR	+	+	NR
	IL-23	+	NR	NR	NR	NR	NR
	IL-24	NR	+	NR	NR	NR	NR
	IL-33	NR	+	NR	+ +	NR	NR
	IL-35	NR	NR	NR	NR	NR	NR
	IFN-γ/TNF-α	+ +	+	NR	+	+	+ + +
	ROS	NR	NR	NR	+	+ +	NR
	Chemokines	+ +	+ +	+	+ + +	+ +	+
Pathways	PI3K/AKT	+	NR	NR	NR	NR	+
	JAK/STAT	+	+	NR	NR	NR	+
	NF-κB	+	NR	NR	NR	+	NR
	ERK/MAPK	NR	+ +	NR	NR	+	NR
	mTOR	NR	NR	+	NR	NR	NR
	HIF-α	NR	NR	+	NR	NR	NR
	Ferroptosis	+	NR	+	NR	NR	NR
Mouse strain	BALB/c	+ + +	+ + +	+ + +	+ + +	+ + + +	+ +
	C57BL/6	+ + + +	+ + +	+ + +	+ + +	NR	+ +
Mouse age	4–6 weeks	+	+	NR	+	NR	+
	6–8 weeks	+ + + +	+ + + +	+ + + +	+ + + +	+ + + +	+ +
	8–12 weeks	+	+	NR	NR	NR	NR
	> 12 weeks	NR	NR	NR	NR	NR	+
Sex	Male	+	+	+	+ +	+	+
	Female	+ + + +	+ + + +	+ + + +	+ + +	+ + + +	+ + +
Duration	2–4 weeks	+ + + +	+ + + +	+	+ + +	NR	+
	4–6 weeks	+	+	+ +	+ + +	+ + + +	+
	6–8 weeks	+	NR	+ +	NR	NR	NR
	> 8 weeks	NR	NR	+ + +	+	NR	+ +
References		Kim et al. 2020; Wen et al. 2024; Quoc et al. 2023; Liu et al. 2024; Jia et al. 2024; An et al. 2022; Pan et al. 2021; Zhang et al. 2020; Camargo et al. 2017; Dejager et al. 2015; Zhang et al. 2015; Zhang et al. 2017; Lee et al. 2017; Park et al. 2022; An et al. 2018; Han et al. 2024; Zhang et al. 2024; Zhang et al. 2022; Immormino et al. 2022; Shilovskiy et al. 2024; Liao et al. 2024)	Kwak et al. 2021; Tage et al. 2024; Jung et al. 2021; Volder et al. 2023; Khalil et al. 2025; Ma et al. 2021; Feng et al. 2022; Namakanova et al. 2022; Duan et al. 2023; Allam et al. 2023)	Li et al. 2020; Hao et al. 2015; Tao et al. 2025; Belvisi et al. 2018; Liu et al. 2025; Li et al. 2021)	Curren et al. 2023; Messaoud-Nacer et al. 2025; Hossain et al. 2020; Nguyen et al. 2016; Rajput et al. 2018; Mahmutovic Persson et al. 2018)	Kim et al. 2017; Zhang et al. 2023; Essilfie et al. 2012; Zhang et al. 2020; Wang et al. 2019)	Tashiro et al. 2017; Mohamed And Amrani 2024; Wang et al. 2023)

Comparison of major NA mouse models via immune cells, mediators, pathways, pathological features, and translational applicability, offering a concise framework for selecting models according to mechanistic or therapeutic research goals

*Abbreviation*: *CS* cigarette smoke, *DC* dendritic cell, *EOS* eosinophil, *ERK* extracellular regulated protein kinases, *HDM* house dust mite, *HIF-α* hypoxia-inducible factor-α, *IFN-γ* interferon-γ, *IL* interleukin, *JAK* Janus kinase, *MAPK* mitogen-activated protein kinase, *Mφ* macrophage, *NEU* neutrophil, *NF-κB* nuclear factor kappa-B, *OVA* ovalbumin, *PI3K* phosphatidylinositol 3-kinase, *AKT* Protein Kinase B, *mTOR* mammalian target of rapamycin, *ROS* reactive oxygen species, *STAT* signal transducer and activator of transcription, *TH1* T helper cell 1, *TH17* T helper cell 17, *TNF-α* tumor necrosis factor-α, *NR* not reported

+ low prevalence

+ +, moderate prevalence

+ + +, high prevalence

+ + + +, very high prevalence

To enhance mechanistic confidence and translational relevance in NA research, in vivo and in vitro systems should be strategically integrated. This begins by aligning model choice with the research question and linking systems through shared pathogenic stimuli, such as bacterial products, CS, and cytokine mixtures, ensuring that epithelial responses in ALI cultures, organoids, or lung‑on‑a‑chip are elicited under conditions comparable to the chosen in vivo protocol. Harmonizing key functional endpoints, including neutrophil recruitment/transmigration, chemokine/cytokine profiles, barrier integrity, corticosteroid responsiveness, and pathway signatures, enables direct cross‑platform comparison. Mechanistic validation can be achieved via parallel perturbation experiments, employing matched inhibitors, neutralizing antibodies, or genetic tools in murine models to test whether a molecular node consistently controls cellular phenotypes in vitro and pathophysiology in vivo. Furthermore, complementary species address different scales and chronicity: mice for pathway dissection, rats for steroid hyporesponsiveness, equine models for chronic remodeling. Ultimately, cross-platform consistency should serve as an explicit criterion for model selection and target prioritization, favoring mechanisms that are reproducibly observed across multiple systems.

Despite these opportunities, critical limitations still remain. Most animal models are acute and fail to recapitulate the chronicity of human NA. Substantial variability in stimuli preparation, challenge protocols, and cell culture methods, exemplified by HDM models, where extract source, batch potency, endotoxin contamination, and handling protocols affect reproducibility, complicates cross‑study comparisons and clinical extrapolation. Additionally, the lack of reliable biomarkers for patient stratification, such as sputum IL‑17, NETs‑associated proteins, inflammasome-derived IL-1β, circulating tight junction fragments, or HDAC2 activity, hampers clinical trial design. To address these gaps, future research should prioritize several strategic directions. First, there is an urgent need to develop and validate standardized modeling protocols that reduce inter-labor variability and improve reproducibility. Second, greater emphasis should be placed on establishing chronic NA models that incorporate extended timelines, repetitive challenges, and longitudinal readouts to better mimic human disease progression. Finally, enhancing the clinical relevance of these models through the incorporation of patient-derived biological materials, comorbid factors (e.g., obesity, infection), and realistic environmental exposures will be essential to bridging the current divide between basic mechanistic research and the development of effective therapies for NA. Moreover, future therapeutic development will increasingly target pathway‑specific nodes identified through the models described above, and will depend on biomarker‑guided patient stratification and rational combination therapies tailored to individual endotypes, for example, targeting IL‑17 together with neutrophil elastase, or inhibiting PI3K while activating HDAC2. By aligning model selection with specific research questions and strategically integrating complementary systems, from high‑throughput murine screens to human cell‑based validation, the field can accelerate the translation of NA‑targeted therapies from bench to bedside.

## Data Availability

No datasets were generated or analysed during the current study.

## Abbreviations

AECs: Airway epithelial cells
AHR: Airway hyperresponsiveness
ALI: Air–liquid interface
Alum: Aluminum hydroxide
BALF: Bronchoalveolar lavage fluid
CFA: Complete Freund’s Adjuvant
CS: Cigarette smoke
CXCL: C-X-C motif chemokine ligand
CXCR: C-X-C chemokine receptor
DCs: Dendritic cells
HDAC2: Histone deacetylase 2
HDM: House dust mite
HFD: High fat diet
IFN-γ: Interferon-gamma
IL: Interleukin
LPS: Lipopolysaccharide
NETs: Neutrophil extracellular traps
NETosis: Neutrophil extracellular trap formation
NA: Neutrophilic asthma
NTHi: Non-typeable Haemophilus influenzae
OVA: Ovalbumin
RSV: Respiratory syncytial virus
RV: Rhinovirus
TGF-β: Transforming growth factor-beta
Th: T helper
T2: Type 2
TLR: Toll-like receptor
TNF-α: Tumor necrosis factor-alpha
